# Supplementary material for: Role of thyroid autoimmunity independent of thyroid hormone levels in pulmonary function decline: A population-based study in euthyroid adults
Source: PLoS One. 2025 Dec 2;20(12):e0336920. doi: 10.1371/journal.pone.0336920 (PMC12671791; doi:10.1371/journal.pone.0336920)
Supplement: Attachment — Submitted filename: Response to reviewers.docx [file pone.0336920.s001.docx]

**Response to reviewers**

**Reviewer 1**

**Comment 1:** The authors should correct the references. The References should be in Journal Format.

**Response to comment 1:** All references have been thoroughly revised and reformatted according to the journal’s reference style requirements.

**Comment 2**. The authors have mentioned that this study complied with the ethical standards of the Declaration of Helsinki and was approved by the Catholic University of Korea,

Catholic Medical Center, Eunpyeong St. Mary’s Hospital Institutional Review Board

(approval no. PC25ZISI0065). Mention the Intuitional Ethical Clearance date also.

**Response to comment 2.:** We have now included the date of institutional ethical approval in the revised manuscript. The sentence has been updated as follow:

Eunpyeong St. Mary’s Hospital Institutional Review Board (approval no. PC25ZISI0065, April 10^th^ 2025).

**Reviewer 2.** This study explored the relationship between thyroid function and lung performance in euthyroid Korean adults. Using data from over 2,600 participants aged 40 and above, the researchers measured lung capacity and airflow with spirometry tests. They analyzed thyroid hormone levels and the presence of thyroid peroxidase antibodies (TPOAb), adjusting for lifestyle and demographic factors. The results showed that higher free thyroxine correlated with better lung function, while higher TSH related to poorer outcomes. Importantly, elevated TPOAb levels were linked to reduced lung function, independent of hormone levels. These associations were stronger in older adults and those with lower body mass index. The findings suggest that even without overt thyroid disease, thyroid autoimmunity can impair pulmonary health. The study addressed an underexplored correlation between thyroid autoimmunity and lung function. While the findings are not yet clinically applicable, they provide valuable insight and contribute to the literature, highlighting the need for further research to clarify and establish potential clinical relevance.

**Response to comment:** We sincerely thank the reviewer for the insightful feedback.
